# The Safety of Micro-Implants for the Brain

**DOI:** 10.3389/fnins.2021.796203

**Published:** 2021-12-09

**Authors:** Abdel-Hameed Dabbour, Sheryl Tan, Sang Ho Kim, Sarah-Jane Guild, Peter Heppner, Daniel McCormick, Bryon E. Wright, Dixon Leung, Robert Gallichan, David Budgett, Simon C. Malpas

**Affiliations:** ^1^Auckland Bioengineering Institute, University of Auckland, Auckland, New Zealand; ^2^Centre for Brain Research, Department of Anatomy and Medical Imaging, University of Auckland, Auckland, New Zealand; ^3^Auckland Bioengineering Institute, Department of Physiology, University of Auckland, Auckland, New Zealand; ^4^Auckland District Health Board, Auckland, New Zealand

**Keywords:** micro-implant, microdevice, implant migration, brain implant, micro-implant safety, micro-implant GFAP, micro-implant IBA-1

## Abstract

Technological advancements in electronics and micromachining now allow the development of discrete wireless brain implantable micro-devices. Applications of such devices include stimulation or sensing and could enable direct placement near regions of interest within the brain without the need for electrode leads or separate battery compartments that are at increased risk of breakage and infection. Clinical use of leadless brain implants is accompanied by novel risks, such as migration of the implant. Additionally, the encapsulation material of the implants plays an important role in mitigating unwanted tissue reactions. These risks have the potential to cause harm or reduce the service of life of the implant. In the present study, we have assessed post-implantation tissue reaction and migration of borosilicate glass-encapsulated micro-implants within the cortex of the brain. Twenty borosilicate glass-encapsulated devices (2 × 3.5 × 20 mm) were implanted into the parenchyma of 10 sheep for 6 months. Radiographs were taken directly post-surgery and at 3 and 6 months. Subsequently, sheep were euthanized, and GFAP and IBA-1 histological analysis was performed. The migration of the implants was tracked by reference to two stainless steel screws placed in the skull. We found no significant difference in fluoroscopy intensity of GFAP and a small difference in IBA-1 between implanted tissue and control. There was no glial scar formation found at the site of the implant’s track wall. Furthermore, we observed movement of up to 4.6 mm in a subset of implants in the first 3 months of implantation and no movement in any implant during the 3–6-month period of implantation. Subsequent histological analysis revealed no evidence of a migration track or tissue damage. We conclude that the implantation of this discrete micro-implant within the brain does not present additional risk due to migration.

## Introduction

Brain implantable technologies, such as neurostimulators, are a rapidly progressing research area. They are used to treat and manage a variety of conditions such as Parkinson’s Disease, treatment-resistant depression, and epilepsy (DeGiorgio et al., 2000; Ben-Menachem, 2002; Bewernick et al., 2010; Fasano et al., 2010; Okun, 2012; Taghva et al., 2013; Fisher and Velasco, 2014; Bergfeld et al., 2016). Traditionally these neural implants require bulky infraclavicular implanted power supplies and electronics. These power supplies require long leads to connect to the implant which are prone to becoming infected or breaking (Oh et al., 2002; Sillay et al., 2008; Mackel et al., 2020). Technological advancements in miniaturization, self-powering and wireless power transfer have allowed for the emergence of discrete wireless micro-implants that no longer need internal batteries or leads (Carrera et al., 2009; McCall et al., 2013; Ahmed and Kakkar, 2017; Beker et al., 2017; Khalifa et al., 2017; Lee et al., 2017; Chen et al., 2018; Feng and Constandinou, 2018). These innovations provide an avenue for reducing hardware-related failures and an option for discrete placement at the site of interest.

Several discrete brain implants are currently in development, with functions varying from neuronal sensing and stimulating to pressure measurement (Guenther et al., 2009; McCall et al., 2013; Benabid et al., 2019; Mitchell et al., 2020). Confidence in their safety has specific challenges and their long-term clinical use comes with novel risks. The risk of migration of the implant from its intended position could cause neurological damage or reduce the efficacy of therapy and must be considered. To our knowledge, no published study has attempted to preemptively quantify the effect or distance of migration of a discrete brain micro-implant. Additionally, neural tissue interaction with the implant material over time could cause neurotoxicity or other damage (Gulino et al., 2019). Historically, implant casings have been metallic (Chong et al., 2020). Borosilicate glass encapsulation is an attractive alternative to metallic enclosures due to its excellent material properties, biocompatibility and recent advancements in micro-machining (Mund and Leib, 2004; Ginggen et al., 2008; Hansen et al., 2009; Leib et al., 2009).

A reduction in infection rates and hardware failures by using a wireless implant may improve service life of the implant and reduce infection rates. In the present study, we have implanted borosilicate glass encapsulated micro-implants into the cortex of sheep for 6 months and assessed the histological tissue response and the potential for implant migration.

## Materials and Methods

Twenty implants were constructed from borosilicate glass with dimensions of 20 × 3.5 × 2 mm. Each implant included a hole of 0.8 mm diameter at one end. In 10 out of the 20 implants, a polyamide non-absorbable monofilament suture (Dafilon^®^ 2/0 DS24) was threaded through the hole and tied in a loop approximately 2 cm long. This was done as a potential measure to facilitate post-operative localization.

Each of the glass implants had an internal cavity suitable for housing device electronics. For this study, these cavities were hermetically sealed and contained non-functional electronic components (PCB, copper wire in a coil) and silver epoxy. Only the external borosilicate glass surfaces of the implant contacted tissue. These internal components were used to regulate density at approximately 2.1 g/cm^3^ to emulate a functioning implant’s density and increase contrast in radiograph images. Device density was approximately twice that of brain tissue (Barber et al., 1970). All implants were sterilized with ethylene oxide before implantation.

**FIGURE 1:**
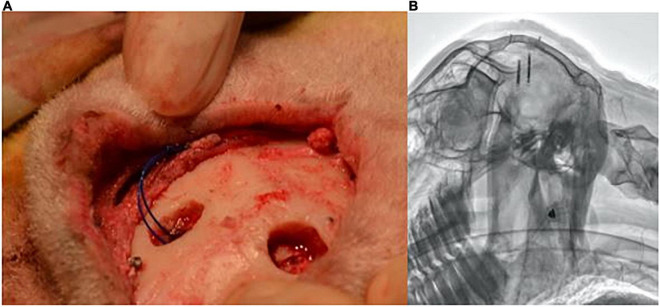
Burr holes’ location and subsequent lateral radiograph. **(A)** Positioning of burr holes and implant location in reference to the skull’s sutures. **(B)** Example lateral radiograph produced from the implant locations.

All experiments were approved by the University of Auckland Animal Ethics Committee. Under general anesthesia, ten female sheep (53.8–64.4 kg) had two implants inserted, one into each hemisphere of the cerebral cortex: one of which had a locator thread. Anesthesia was induced with Propofol (2–5 mg/Kg i.v.) and maintained by (2–3%) isoflurane after intubation and ventilation. Antibiotics (2 mg/kg I.V. ceftiofur sodium in sterile water) and analgesia (2 mg/kg I.M. Keptoprofen) were also given along with long-acting local anesthetic (2.5 mg/kg bupivacaine) used at the skin incision site. Under sterile conditions, a midline skin incision was made on the top of the head, and an approximately 10 mm burr hole was placed 10 mm from the sagittal and lambdoid sutures. A second burr hole was created on the opposite side of the sagittal suture in a staggered position such that the implants did not overlay each other in radiographs. The dura and pia were cut, and the implants were inserted approximately 1 mm below the cortex using a tool designed to hold and insert the implants. Figure 1A displays the location of the implants and burr holes post-insertion. Two surgical screws (3–4 mm in length) were rigidly fixed into the skull next to the burr holes as reference points for the radiographs. A set of radiographs were taken and analyzed post-surgery, at 3 month follow up and at a 6 month follow up.

### Tissue Collection and Preparation

Following the 6-month radiographs, the sheep were euthanized. The carotid artery was exposed, and a cannula was inserted, allowing the tissue to be flushed with 0.9% saline solution. Cutting of the jugular vein allowed for the drainage of blood and excess saline. Brains were then perfused with 10% formalin and extracted from the skull. The brain was divided into left and right hemispheres following removal of the dura mater and meninges and post-fixed in 10% formalin for 72 h. Brain tissue was briefly rinsed in phosphate buffered saline (PBS) and transferred to 70% ethanol prior to further dissection and processing for embedding in paraffin wax, as described by Zapiec et al. (2017).

### Fluorescent Immunohistochemistry

Following tissue processing, tissue blocks containing the region where the implant was inserted, as well as a control from an unaffected cortical region from the same sheep were cut in a sagittal orientation, enabling the entire longitudinal-section of the implant to be analyzed. 10 μm thick sections were subject to heat-induced epitope retrieval with 10 mM sodium citrate buffer, pH 6.0 (GFAP and collagen IV) or citric acid buffer, pH 6.0 (IBA-1), blocking, and antibody incubations using a protocol adapted from Zapiec et al. (2017). Rabbit polyclonal, primary antibodies used were: glial fibrillary acidic protein (GFAP), 1:1,000 (Dako, Z0334), ionized calcium-binding adapter protein (IBA-1), 1:500 (Wako 019-19741), and collagen IV (Col4A), 1:300 (Biorbyt 340147). Goat anti-rabbit IgG (H+L) Alexa Fluor 647 was used as the secondary antibody for single-label fluorescent immunohistochemistry and Hoechst 33342 (Molecular Probes, H1399), 1:10,000, as a nuclear counterstain.

### Imaging and Processing

Slides were scanned using the VSlide scanner (Metasystems, Althussheim, Germany) operating with a Zeiss AxioImager Z2 using a Plan Apochromat 10X/0.45 objective (Zeiss AG, Jena, Germany). Images were obtained with a CoolCube 4mTEC monochrome sCMOS camera (Metasystems, Althussheim, Germany) and stitched with Metafer (version 5). All parameters related to the operation of the slide scanner were optimized and recorded as classifiers; settings for wavelength, exposure, focal distance, and magnification were stored for each label, ensuring a consistent, standardized imaging workflow across the entire dataset. The stitched images were opened in VSViewer v2.1.133 (Metasystems, Althussheim, Germany), in which the individual fields of view comprising the entire stitched image were numbered. Six 4,096 × 3,000 pixel fields of view (corresponding to a total area of 1.420 mm × 6.241 mm on either side of the implant) were extracted for analysis. An equivalent area was selected in the internal/uninjured control blocks. Macros for background subtraction, thresholding, and measurement of mean gray values (as an index of fluorescence intensity) for the selected fields of view were optimized and run in Fiji v1.53J (Schindelin et al., 2012).

### Histology Statistical Analysis

Statistical analysis was performed in GraphPad Prism v9.0.2 (SmartDrawNet, San Diego, United States). A *p*-value greater than 0.05 (*p* ≥ 0.05) was considered statistically significant. For each label, mean gray values (MGV) from left and right hemispheres were combined to obtain a single value for each animal; the same was performed for the internal controls. Unpaired *t*-tests comparing implanted and uninjured tissue were performed for both absolute MGV and as a function of area (MGV/mm^2^).

### Radiograph Analysis

The positions of the implants were tracked using lateral radiographs. A lateral radiograph was taken following the implantation surgery, and subsequent follow-up radiographs were taken 3 months and 6 months after the surgical procedure.

The radiographs were taken at 50 cm away from the X-ray plate. The first radiographs were taken with the sheep still under anesthesia; at the 3-month and 6-month radiographs, the sheep were lightly sedated using Acepromazine (0.04 mg/kg I.V.) to allow for correct positioning of the head.

An example radiograph can is shown in Figure 1B. The two implants can be seen alongside the two reference screws. Analysis of each implant’s displacement was conducted using the Asteris Keystone Omni software. The known nominal diameter of one of the reference screws was used to calibrate the images.

**FIGURE 2:**
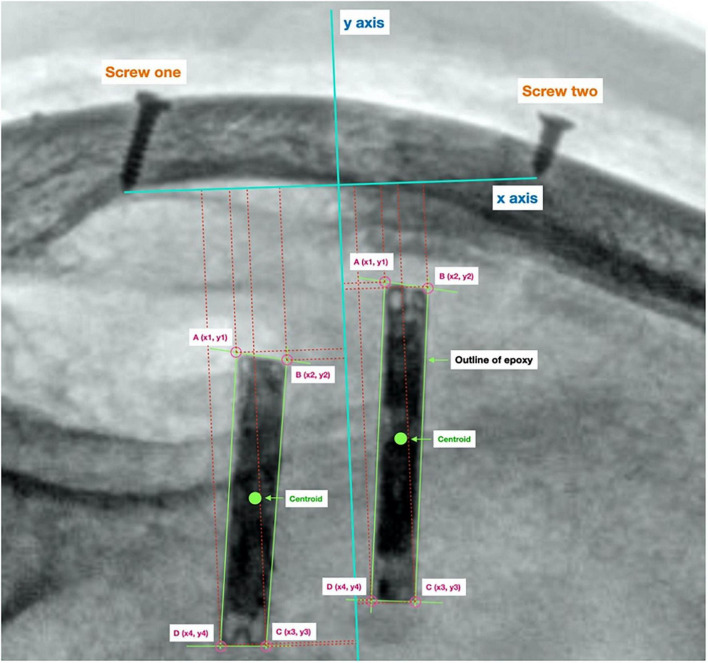
Reference origin used to calculate displacement. Origin created using two screw’s lodged in the sheep’s skull, used to track the location of the centroid of each implant between radiographs.

A 2D reference coordinate system was created to measure the displacement of the implants. The origin was taken to be the midpoint between the tips of the two reference screws (*x*-axis). The *y*-axis was taken to be perpendicular to the origin. This can be seen in Figure 2.

The coordinates of each of the four corners of each implant were measured relative to the origin. These coordinates were then used to calculate the centroid coordinate of each implant. The centroid displacement between two different radiographs was calculated using the formula below, the x coordinate of the prior position (in the previous follow up radiograph) of the implant’s centroid is denoted by *x*_1_, while the current x coordinate is denoted by *x*_2_. Similarly, the y coordinate of the prior position of the implant’s centroid is denoted by *y*_1_, and the current y coordinate is denoted by *y*_2_.


$$C\,e\,n\,t\,r\,o\,i\,d\,D\,i\,s\,p\,l\,a\,c\,e\,m\,e\,n\,t=\sqrt{{\left(x_{2}-x_{1}\right)}^{2}+{\left(y_{2}-y_{1}\right)}^{2}}$$

To estimate the impact of the inevitable variability in the positioning of the sheep’s head at different time points, amongst other factors of measurement variation, the Measurement Detection Limit (MDL) was calculated using the mean difference of the length of the *x*-axis made between groups of repeated scans for the same sheep at two head positions. The MDL was calculated to be 2.4 mm, i.e., movement of the implant>2.4 mm would have been detectable.

A *P*-value of greater than 0.05 (*P* > 0.05) was used to determine significance in the statistical methods used. The normality of the migration was tested using the Anderson-Darling method. Two-sample *t*-tests were used to determine differences of mean migration between 0–3 and 3–6 months by performing a two-sample *t*-test.

## Results

**FIGURE 3:**
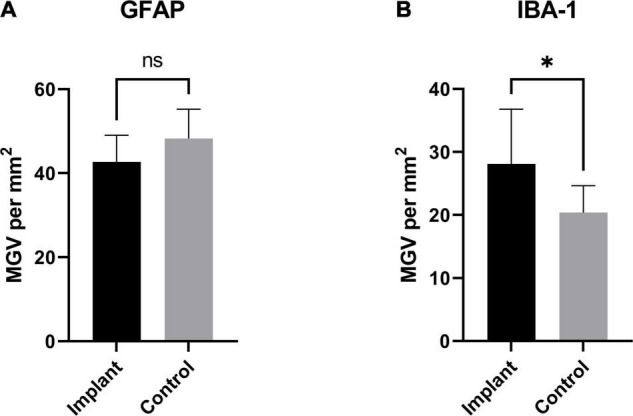
GFAP and IBA-1 expression as a function of area: **(A)** GFAP expression (measured as total mean gray value per mm^2^) from the implanted samples compared to an equivalent area from an un-implanted region of sheep brain. While GFAP was slightly decreased in the implanted tissue, this difference was not significant (*p* < 0.1131). The same measurements were made to assess the expression of IBA-1 in implanted tissue and an equivalent area from and un-implanted region **(B)**. There was a small, significant increase in the implanted tissue compared to un-implanted control (*p* < 0.0419). * Indicates a statistically significant difference.

**FIGURE 4:**
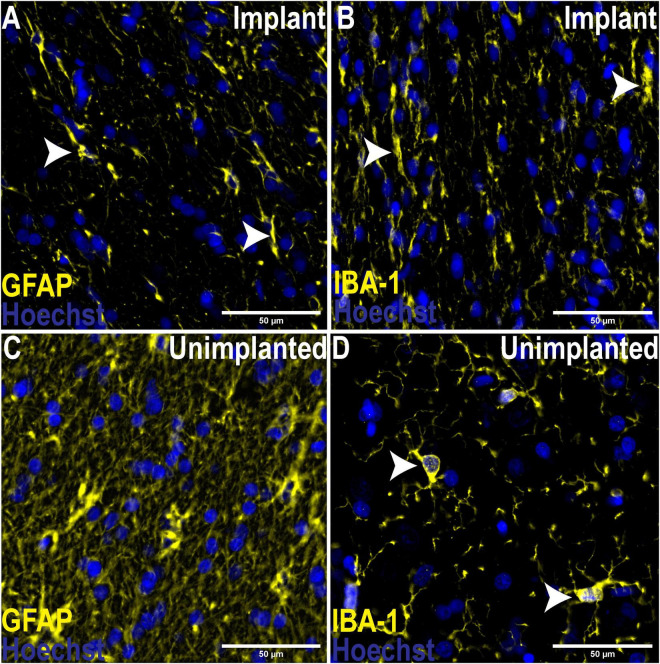
Comparison of morphological changes of astrocytes and microglia adjacent to the implant track and un-implanted tissue. 20X Magnification. **(A)** Astrocytes adjacent to the implant track assumed directionality and processes were diminished in contrast to un-implanted tissue **(C)**, where astrocytes displaced their classic, stellate morphology with numerous processes extending in all directions of the microenvironment. Like astrocytes in implanted tissue, microglia also assumed directionality and processes appeared thicker **(B)**, indicated an activated phenotype compared to their quiescent state in the absence of trauma **(D)**, where microglia assume a rounded morphology with thin, stellate-like processes.

To assess the foreign body response to the implant we analyzed the expression of GFAP, a marker of activated astrocytes, and IBA-1, which detects microglia, two key markers of scar formation/encapsulation and inflammation (Polikov et al., 2005). Fluorescence intensity, expressed as mean gray values (MGV), was used as an index of expression. In comparison to an equivalent area of uninjured brain parenchyma from the same sheep, there was no significant increase in GFAP expression (Figure 3A; 42.67 ± 2.244/mm^2^ implanted vs. 48.29 ± 2.451/mm^2^ un-implanted; *p* < 0.1131). Qualitatively, examination of the astrocytes at higher magnification (20X) showed that while the morphology of cell bodies in both implanted and un-implanted tissue remained similar, astrocytic processes are markedly diminished in the former which is likely to account for the small, statistically insignificant decrease in GFAP immunoreactivity compared to the un-implanted tissue (Figures 4A,C, white arrows). Furthermore, processes in the implanted tissue appeared elongated and thin, whilst astrocytes in uninjured tissue retained their classic, stellate morphology with processes extending in all directions of their microenvironment, including in and out of the sectioned tissue plane. Conversely, a modest yet significant difference was observed with microglial expression (Figure 3B; 28.07/mm^2^ ± 3.083 implanted, 20.38/mm^2^± 1.515 un-implanted, *p* < 0.0419). A qualitative examination of the microglia near the implant track indicated that these cells remain in a persistent state of activation; cellular processes appeared thicker and assumed directionality in the same plane as the implant (Figure 4B; white arrows). In contrast, microglia in the un-implanted control retained the canonical, quiescent morphology characterized by long, thin, stellate-like processes.

After 3 months of implantation, we observed that six of the twenty implants showed signs of minor movement greater than our MDL. This movement was between 2.5 and 4.4 mm. All other implants showed no sign of movement. After a further 3 months of implantation (6 months post-implant insertion), no implants showed evidence of movement larger than the MDL [mean absolute displacement (1.3 ± 0.6 mm, std dev)]. Two sheep were excluded from analysis due to the loss of a reference screw. There was no evidence of significant rotation of the implant throughout both series of measurements. Both populations of migration data (0–3 months and 3–6 months) were normally distributed (*P* > 0.05). A two-sample *t*-test found the difference in the means of the two groups to be 0.79 (95% CI [0.059, 1.522]). No statistical difference was found in the mean value of movement between the implants with a locator thread and without.

## Discussion

Currently, implantable stimulators and pressure sensors have a form-factor of an electrode/sensor within the cortex connected via a cable to a central implant often placed under the skin on the chest or to an external processor. In deep brain stimulation systems, hardware-related infections have been reported to be as high as 23%, and associated with significant morbidity (Fily et al., 2011). Studies have also shown lead breakages to be a cause of loss of DBS efficacy, and reduced tremor control and paresthesia (Kondziolka et al., 2002). Therefore, there is a substantial opportunity for improving clinical outcomes by having a discrete implant performing all the functions required. The present study details the implantation of borosilicate glass encapsulated micro-implants into cortical sheep brain tissue. Overall, we observed no evidence of a migration track, tissue damage or glial encapsulation after 6 months of implantation.

The implants used in this study have an outer case composed solely of borosilicate glass. Borosilicate glass has been proposed as a biocompatible housing for an intracranial pressure measuring implant (Ginggen et al., 2008). Previous studies conducted in rats have also showed that borosilicate glass performed better on measures of inflammation, neuronal loss, and hemorrhaging when compared to sapphire (Parthasarathy et al., 2007). Borosilicate glass is also used as a control in biocompatibility studies (Silver et al., 2001; Price et al., 2003). Although borosilicate glass has been shown to be biocompatible, some response in the surrounding tissue is an inevitable consequence of introducing a foreign body into the brain.

**FIGURE 5:**
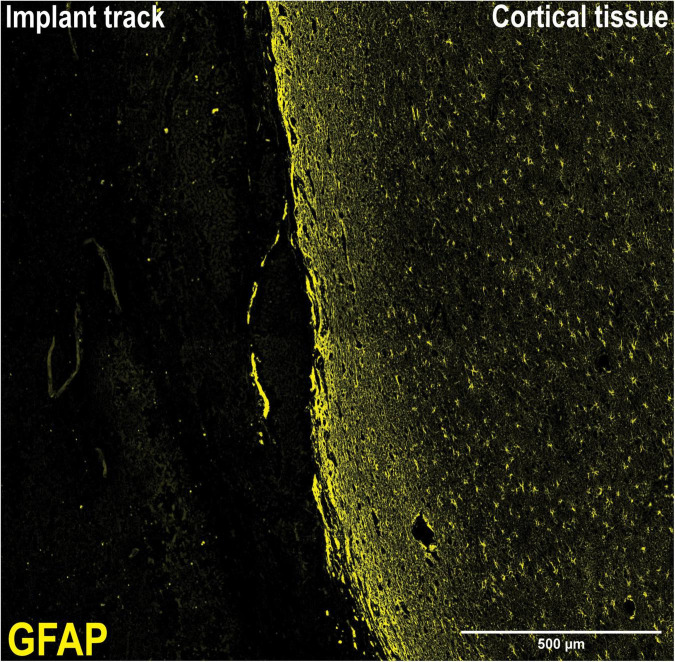
10X Magnification of implant track. Lack of a visible glial scar at the tissue-implant interface necessitated a different approach to analysis. At the tissue-implant interface, there was no discernible visible scar tissue to measure, necessitating the measurement of fluorescence intensity from a given area either side of the implant track, supplemented with morphological analysis.

The duration of implantation reflects a chronic timeframe where implants are left in the brain for a number of months, with the potential to be left *in situ* over the course of a lifetime. This time period is characterized by substantial tissue re-modeling as the surrounding tissue attempts to protect itself from the foreign body. Steady-state of tissue remodeling is said to have occurred at 12 weeks (Prodanov and Delbeke, 2016). We measured astrogliosis and microglial activation; two key indices of the foreign body response at the long-term/chronic time frames (Polikov et al., 2005; Prodanov and Delbeke, 2016). The histological study performed yielded similar results on all sheep. There was no substantial glial thickening surrounding the implant track wall (Figure 5), which was unexpected given that this has been reported as being prominent in other models of neural implantation (Szarowski et al., 2003; Salatino et al., 2017). A review on implant design suggests that implants with closer density to that of brain tissue will display less glial scarring. In addition, untethered wireless implants will experience less tissue reaction than tethered implants (Prodanov and Delbeke, 2016). Tethered implants such as DBS probes act as cantilever beams within the brain, as such if they possess high stiffness and are not able to flex with the micromotions of the brain they will induce higher tissue damage, response and glial scarring (Stiller et al., 2018). A wireless implant that is able to be discretely implanted at the site of interest eliminates this risk. Although minor migration away from the intended position may reduce the efficacy of stimulation. Optogenetics, an emerging method of neural stimulation, offers advantages to traditional probe neural stimulation. Stimulation via optical methods allows for specific neuronal stimulation. Wireless optogenetic implants intended to overcome the issues of traditional DBS are in development (Kim et al., 2013).

In our study, GFAP immunoreactivity was slightly diminished compared to an un-implanted region (Figures 4A,C); however, this difference was not significant (Figure 3A; *p* < 0.1131). This observation is consistent with previous studies in rats where analysis of GFAP at equivalent “chronic” time points (3–9 months) indicated very little astrogliosis (Mokrý et al., 2000; Szarowski et al., 2003; Polikov et al., 2005). Further, astrocytic processes appeared to assume directionality alongside the implant track compared to the astrocytes located in an un-implanted region, where processes extended in all directions and planes as part of surveying the immediate microenvironment. The observed directionality suggests a foreign body response has taken place; however, at 6 months following implantation it appears that the scar tissue formation process was no longer active. This is further bolstered by the lack of collagen IV immunoreactivity around the implant track which was confined primarily to the microvasculature where it is normally expressed and serves as an integral structural component. Minimal astrogliosis has also been reported in patients diagnosed with Parkinson’s disease who had received long-term implants as part of Deep Brain Stimulation (DBS) therapy (Haberler et al., 2000), as well as in rodent studies using microelectrode implants (Pflüger et al., 2020). Conversely, a modest yet statistically significant microglial response was seen in the present study. IBA-1 expression was higher in the tissue near the implant, compared to a region of equivalent size well away from the implant size (Figure 3B). Critically, the increase is small and not pronounced, indicating that microglial activity is only minimally elevated. Again, microglial processes appeared thicker and assumed directionality with the implant track when examined at higher magnification (Figure 4B), a stark contrast to the quiescent morphology exemplified in Figure 4D. This is characteristic of “frustrated phagocytosis,” where a foreign body response is mounted, but microglia are unable to engulf and digest the material they intend to remove (Rogers et al., 2002). Initially observed in neurodegenerative disease to describe an inability of microglia to clear β-amyloid plaques (Rogers et al., 2002), this has also been observed in rat brains implanted with microelectrode arrays (Biran et al., 2005). Consequently, microglia remains constitutively active, enhancing the section of inflammatory molecules that have neurotoxic potential (Rogers et al., 2002). They will not be as reactive to a borosilicate glass implant, compared to another foreign body such as β-amyloid. Overall, these results indicate that the biological response to borosilicate glass was very minor and would be unlikely to result in changes in surrounding neuronal function.

There have been few studies that have explored foreign body migration through the brain. Predominantly these have been in the form of metallic fragments such as shrapnel or bullets, which have been shown to move up to 10 mm a day (Kenneth et al., 1976; Pelin and Kaner, 2012; Negrotto et al., 2019; Hammed et al., 2021). The potential risks of foreign body migration have been described as infection, ventricular obstruction causing hydrocephalus, and neurological deficits as a consequence of migration. In the present study, we found minimal movement, up to 4.6 mm, of our small, light implants over the 6 months. We believe the significantly smaller movement we observed could be due to the density of our implant (2.1 g/cm^3^) being close to the density of brain tissue of 1.05 g/cm^3^ (DiResta et al., 1991). We anticipate a wireless implant cased in a metallic housing such as titanium (density 19.25 g/cm^3^) or stainless steel (density 8 g/cm^3^) to experience larger migration evoking a larger tissue response than seen in this study. Additionally, implant geometry is expected to have an impact on the movement, thin implants or implants with sharp edges that are able to sheer the parenchymal tissue would be expected to migrate and illicit a larger tissue response than seen in this study. The extent of the impact of density and geometry on migration is not well understood and would be suited for future works in the area of wireless micro-devices for brain implantation.

**FIGURE 6:**
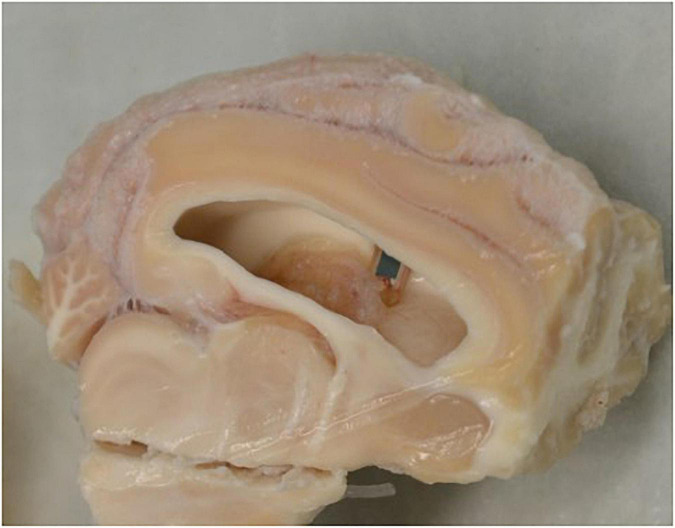
Section of brain tissue displaying the implant piercing the ventricle wall. Dissection of brain tissue following the sacrifice of the sheep outlining the piercing of the ventricle wall by an implant.

We observed in four sheep that the end of an implant pierced the ventricle; this can be seen in Figure 6. This was an inevitable outcome in some cases given that the cortical thickness at the site of implantation varied between 12 and 24 mm. A past case study on foreign body migration indicated that movement tends to occur in the direction of gravity (Kenneth et al., 1976). As such, it was expected for any significant migration to have occurred in the direction of the longest axis of the implant and would have been observable in the lateral radiographs. This is the basis for analyzing the lateral radiographs. We noted that in no cases had the implant migrated into the ventricle. We propose that this is additionally supportive that the implants were not migrating through the cortex.

We used single plane radiographs at 3-time points (0, 3, and 6 months) as the primary means to test for movement of the implant. As indicated in the results this technology does have a measurement error and a future possible approach would be to conduct CT imagery to look for very subtle signs of migration. However, our histological analysis did not support the concept that continual migration was occurring as there was no evidence of a track of migration through the cortex. It would be possible in future studies to undertake histological analysis at shorter time periods e.g., 1–4 weeks to qualify the acute tissue response. Additional histological methods such as Western Blotting or RT-qPCR would also provide a greater understanding of the tissue re-modeling occurring. Finally, we did not have another implant/electrode to act as a reference for the histological analysis. As noted, all foreign bodies will produce some level of host response and it would be useful to compare this response between a wireless micro-implant and an electrode.

The rapid advances in wireless power, communication, and micromachining hold much promise for development of discrete wireless brain implants. Our study has provided the critical knowledge that these glass micro-implants are inherently safe when placed directly within the cortex.

## Data Availability Statement

The raw data supporting the conclusions of this article will be made available by the authors, without undue reservation.

## Ethics Statement

The animal study was reviewed and approved by the University of Auckland Animal Ethics Committee.

## Author Contributions

A-HD, SK, and ST conducted the data analysis. All authors reviewed the manuscript prior to submission and contributed to the design and performance of the study.

## Conflict of Interest

The authors declare that the research was conducted in the absence of any commercial or financial relationships that could be construed as a potential conflict of interest.

## Publisher’s Note

All claims expressed in this article are solely those of the authors and do not necessarily represent those of their affiliated organizations, or those of the publisher, the editors and the reviewers. Any product that may be evaluated in this article, or claim that may be made by its manufacturer, is not guaranteed or endorsed by the publisher.
